# *Arc-*Expressing Accessory Olfactory Bulb Interneurons Support Chemosensory Social Behavioral Plasticity

**DOI:** 10.1523/JNEUROSCI.0847-22.2022

**Published:** 2023-02-15

**Authors:** Kelsey E. Zuk, Hillary L. Cansler, Jinxin Wang, Julian P. Meeks

**Affiliations:** ^1^Neuroscience Graduate Program, University of Texas Southwestern Medical Center, Dallas, Texas 75390; ^2^Department of Neuroscience, University of Rochester School of Medicine and Dentistry, Rochester, New York 14642; ^3^Department of Pharmacology, University of Florida College of Medicine, Gainesville, Florida 32603; ^4^Department of Pediatrics, University of Rochester School of Medicine and Dentistry, Rochester, New York 14642

**Keywords:** accessory olfactory bulb, aggression, Arc, interneuron, plasticity, social behavior

## Abstract

The accessory olfactory system (AOS) is critical for the development and expression of social behavior. The first dedicated circuit in the AOS, the accessory olfactory bulb (AOB), exhibits cellular and network plasticity in male and female mice after social experience. In the AOB, interneurons called internal granule cells (IGCs) express the plasticity-associated immediate-early gene *Arc* following intermale aggression or mating. Here, we sought to better understand how *Arc*-expressing IGCs shape AOB information processing and social behavior in the context of territorial aggression. We used “ArcTRAP” (Arc-CreERT2) transgenic mice to selectively and permanently label *Arc*-expressing IGCs following male–male resident–intruder interactions. Using whole-cell patch-clamp electrophysiology, we found that *Arc*-expressing IGCs display increased intrinsic excitability for several days after a single resident–intruder interaction. Further, we found that *Arc*-expressing IGCs maintain this increased excitability across repeated resident–intruder interactions, during which resident mice increase or “ramp” their aggression. We tested the hypothesis that *Arc*-expressing IGCs participate in ramping aggression. Using a combination of ArcTRAP mice and chemogenetics (Cre-dependent hM4D(G_i_)-mCherry AAV injections), we found that disruption of *Arc*-expressing IGC activity during repeated resident–intruder interactions abolishes the ramping aggression exhibited by resident male mice. This work shows that *Arc*-expressing AOB IGC ensembles are activated by specific chemosensory environments, and play an integral role in the establishment and expression of sex-typical social behavior. These studies identify a population of plastic interneurons in an early chemosensory circuit that display physiological features consistent with simple memory formation, increasing our understanding of central chemosensory processing and mammalian social behavior.

**SIGNIFICANCE STATEMENT** The accessory olfactory system plays a vital role in rodent chemosensory social behavior. We studied experience-dependent plasticity in the accessory olfactory bulb and found that internal granule cells expressing the immediate-early gene *Arc* after the resident–intruder paradigm increase their excitability for several days. We investigated the roles of these *Arc*-expressing internal granule cells on chemosensory social behavior by chemogenetically manipulating their excitability during repeated social interactions. We found that inhibiting these cells eliminated intermale aggressive ramping behavior. These studies identify a population of plastic interneurons in an early chemosensory circuit that display physiological features consistent with simple memory formation, increasing our understanding of central chemosensory processing and mammalian social behavior.

## Introduction

The survival and reproduction of an animal are highly dependent on its ability to appropriately interact with its social environment. Social interactions arise from the integration of complex sensory cues found in the animal's surroundings. More so than humans, terrestrial mammals rely on the detection and interpretation of olfactory stimuli to guide their behavior, both during initial encounters and after experience (Brennan and Keverne, 2015). In rodents, the accessory olfactory system (AOS) plays an essential role in guiding behavior through the detection of chemosignals, which are thought to convey information about the species, sex, reproductive status, or even health of other animals (Brennan, 2001; Salazar and Brennan, 2001; Mucignat-Caretta et al., 2012). Many chemosignals, including pheromones, are detected in the AOS via vomeronasal sensory neurons found in the vomeronasal organ. Vomeronasal sensory neurons are known to be activated by excreted proteins, peptides, and steroids (Leinders-Zufall et al., 2004; Kimoto et al., 2005; Chamero et al., 2007; Nodari et al., 2008; Liberles et al., 2009; Riviere et al., 2009; Wong et al., 2020). Disruption of vomeronasal sensory neuron signaling through knockout of the Trpc2 ion channel has been shown to alter the display of sex-typical behavior, such as mating and aggression, reinforcing the notion that sensory input to the AOS plays a critical role in behavioral output (Stowers et al., 2002). Overall, it is well established that AOS alteration disrupts social behavior, but the circuit-level mechanisms mediating these behavioral changes are still not understood.

Previous work suggested that inhibitory signaling, via GABAergic neurons, plays an essential role in regulating olfactory information processing (Brennan and Keverne, 1997; Jia et al., 1999). Recent studies identified a population of GABAergic inhibitory interneurons in the accessory olfactory bulb (AOB), the first dedicated circuit for AOS information processing, that display increased intrinsic excitability for several hours after social chemosensory experience (Cansler et al., 2017; Gao et al., 2017). These inhibitory interneurons, known as internal granule cells (IGCs), are found in the internal cellular layer (ICL) of the AOB and are the most abundant inhibitory interneuron subtype in this circuit (Larriva-Sahd, 2008). IGCs have small basal dendrites that reside in the ICL and long apical dendrites that stretch into the external cellular layer (ECL), where excitatory mitral cells reside. Each IGC forms reciprocal, dendro-dendritic synapses along the dendrites of multiple mitral cells (Taniguchi and Kaba, 2001; Larriva-Sahd, 2008). These cells are analogous to granule cells in the main olfactory bulb, which have similar morphology, connectivity, and presumably function to their AOB counterparts (Sailor et al., 2016). Main olfactory bulb granule cells demonstrate changes in their dendritic arbors and dendro-dendritic synapses throughout the life of an animal (Sailor et al., 2016; Quast et al., 2017). Experience-dependent structural changes, as measured by electron microscopy, in these dendro-dendritic synapses have also been shown to be associated with olfactory learning in the AOB (Matsuoka et al., 1997). Building on recent work, we sought to further understand how AOB experience-dependent plasticity may be linked to changes in immediate-early gene expression in inhibitory interneurons (Cansler et al., 2017; Gao et al., 2017).

Immediate-early genes, which are quickly and selectively upregulated after a neuronal stimulus response, have been linked to various forms of plasticity (Minatohara et al., 2015). The activity-regulated cytoskeletal-associated gene (*Arc*) has been shown to play a direct role in mediating LTD in hippocampal excitatory neurons (Bramham et al., 2008; Shepherd and Bear, 2011; Jakkamsetti et al., 2013). AOB IGCs that display increased excitability after experience also selectively express *Arc* mRNA and protein (Arc) (Cansler et al., 2017). Arc is associated with experience-dependent plasticity throughout the brain (for review, see Korb and Finkbeiner, 2011), and we hypothesized that *Arc*-expressing IGCs contribute to AOB function in the context of AOS-mediated social behavior.

Here, we report that IGCs that transiently express *Arc* following a single social experience display increased intrinsic excitability for several days. Increased IGC excitability persisted during a week of repeated social experiences but did not scale with the number of experiences. We tested whether *Arc*-expressing IGCs participate in the development of aggressive behaviors between males, an AOS-mediated behavior. We found that chemogenetically inhibiting *Arc*-positive AOB IGCs completely abolished aggressive ramping over multiple intermale resident–intruder interactions. Our results indicate that this early sensory inhibitory neuron population plays a critical role in regulating the behavioral response to social chemosignals.

## Materials and Methods

### Mice

All animal procedures were conducted in compliance with both the University of Texas Southwestern Medical Center Institutional Animal Care and Use Committee and the University of Rochester University Committee on Animal Resources. Sexually naive adult male mice 6-12 weeks of age were used throughout the study. Mice were housed on a reverse 12 h light/dark cycle, with lights on from either noon to midnight or 6:00 P.M. to 6:00 A.M. Food and water were provided *ad libitum*. *B6.129(Cg)-Arctm1.1* (ArcCreERT2^+/−^; The Jackson Laboratory; Stock #021881) were crossed with *Gt(ROSA)26Sortm9(CAG-tdTomato)Hze* (Ai9^+/−^ or Ai9^+/+^; The Jackson Laboratory; stock #007905) (Guenthner et al., 2013). These mice will be referred to as ArcTRAP (ArcCreERT2^+/−^, Ai9^+/−^, or Ai9^+/+^) throughout (Guenthner et al., 2013). In some experiments, the ArcTRAP line was crossed with a hemizygous *Arc-d4EGFP* line (a gift from Pavel Osten, via Kimberly Huber) (Grinevich et al., 2009) to form ArcTRAP × Arc-d4EGFP mice. Each mouse line was maintained on a C57BL/6J background. BALB/cJ mice were obtained from either the University of Texas Southwestern Mouse Breeding Core or The Jackson Laboratory (stock #000651). A total of 207 mice were used in this study.

### Resident–intruder test

All behavioral tests were conducted during the dark cycle. ArcTRAP resident male mice were single housed for 1 week without cage changes. At the end of 1 week, resident mice underwent a process referred to as targeted recombination in active populations (TRAP) (Guenthner et al., 2013), where they were lightly anesthetized with isoflurane (to reduce stress from injection) and intraperitoneally injected with 4-hydroxytamoxifen (4-OHT; Sigma-Aldrich) at a dose of 50 mg/kg. Twenty minutes after 4-OHT injection, a BALB/cJ intruder male was introduced to the resident's cage for 10 min during the resident–intruder test. After 10 min, the intruder was returned to its home cage. Resident mice were left in their home cage and allowed to rest for at least 1 h before being returned to the animal housing facility.

The resident–intruder test was conducted once per day, for up to 8 d. In repeated exposure (re-exposure [RE]) experiments (see Figs. 4–7), resident animals were re-exposed to the same individual BALBc\J intruder for each interaction. Aggressive ramping experiments (see Figs. 6, 7) involved measurements from the same resident–intruder pair each day. All behavior sessions were recorded with handheld cameras (Panasonic) at 30 frames per second. In a subset of our DREADD-manipulated and control mice, behavior videos were recorded from both a side view (used for all manual behavior annotation) and a top view (better suited for DeepLabCut).

### Live slice preparation

Mice were anesthetized with isoflurane and decapitated 1, 3, 5, or 7 d after being TRAPed on day 0. Brains were dissected, and 400 μm parasagittal sections of the AOB were prepared using a vibrating microtome (Leica VT1200) in ice-cold ACSF bubbled with 95% O_2_, 5% CO_2_. Standard ACSF contained the following (in mm): 125 NaCl, 2.5 KCl, 2 CaCl_2_, 1 MgCl_2_, 25 NaHCO_3_, 1.25 NaH_2_PO_4_, 25 glucose, 3 myoinositol, 2 Na-pyruvate, and 0.4 Na-ascorbate (pH 7.4, 315 mOsm). For slicing, an additional 9 MgCl_2_ was added to ice-cold ACSF. After slicing, the slices were kept in a recovery chamber at room temperature (23°C) containing oxygenated ACSF with 0.5 mm kynurenic acid to prevent potential glutamate excitotoxicity during the recovery/holding period (1-6 h). Just before patch-clamp recordings or live 2-photon imaging experiments, slices were transferred to a slice chamber (Warner Instruments) mounted on an upright fluorescence-equipped differential interference contrast microscope (Nikon; model FN1) or 2-photon microscope (ThorLabs Acerra). The tissue was superfused with oxygenated ACSF at 35°C via a peristaltic pump (Gilson) at a rate of 3-3.5 ml/min.

### Electrophysiology

Whole-cell patch-clamp recordings were made on ArcTRAP^+^ (tdTomato^+^) and ArcTRAP^–^ (tdTomato^–^) IGCs 1, 3, 5, or 7 d following TRAP on day 0. Thin-wall borosilicate glass electrodes with a tip resistance between 4.5 and 13.5 mΩ were filled with internal solution containing the following (in mm): 115 K-gluconate, 20 KCl, 10 HEPES, 2 EGTA, 2 MgATP, 0.3 Na_2_GTP, and 10 Na phosphocreatine, pH 7.37. All recordings were amplified using a MultiClamp 700B amplifier (Molecular Devices) at 20 kHz and were digitized by a DigiData 1440 analog-digital converter controlled via pClamp 10.5 software (Molecular Devices; RRID:SCR_011323). Data were analyzed by Clampex 10.5 (Molecular Devices) and custom software written in MATLAB.

Patched AOB IGCs were subjected to a series of current-clamp and voltage-clamp challenges. Immediately after achieving the whole-cell configuration, the resting membrane potential (V_rest_) of each cell was measured in current-clamp mode. To standardize measurements across cells with different V_rest_, we injected steady-state currents to maintain the membrane potential (V_m_) of each cell between −70 and −75 mV. Based on initial measurements of input resistance (R_input_), we empirically determined the amplitude of hyperpolarizing current that adjusted V_m_ by −50 mV (to ∼−125 mV). After determining this initial current injection amplitude, we generated a cell-specific 10 sweep Clampex protocol that applied increasingly depolarizing 0.5 s square current pulses, starting with the initial injection amplitude. For example, if the initial current injection was determined to be −100 pA, the 10 sweep protocol would have current injection increments of 20 pA (i.e., −100, −80, −60, ... 80 pA). In voltage clamp, cells were initially held at −70 mV, and a series of 12 voltage command steps (0.5 s in duration) were applied that spanned −100 to 10 mV. For each cell, both current-clamp and voltage-clamp protocols were applied up to 4 times, and all reported quantities represent the mean responses across repeated trials.

For CNO wash-on experiments, slices were taken from a subset of participants in the behavioral experiments shown in Figure 7*C–H* (*N* = 13 for AAV-Gi-DREADD, *N* = 3 for AAV-mCherry). For each slice, 32 min recording was conducted while measuring baseline resting membrane potential. tdTomato signal was used to identify ArcTRAP^+^ IGCs in slices that contained either the AAV-Gi-DREADD or the AAV-mCherry. In the first 2 min of the recording, ACSF was washed over the slice. In minutes 2-12, 10 μm CNO in ACSF was washed-on. ACSF was then washed over the slice for the remainder of the recording, between minutes 12 and 32. Once exposed to CNO, slices were discarded to avoid possible carryover of CNO effects to subsequent recordings.

### Injectable drug preparation

4-OHT (Sigma; catalog #H6278) was freshly dissolved in warmed ethanol at a concentration of 20 mg/ml on each experimental day. Once dissolved, a volume of corn oil (Acros Organics; catalog #8001-30-7) equivalent to the volume of ethanol was added. The mixture was heated and spun at 1725 rpm in a Speedvac concentrator (Savant; model #SVC-100H) for 20 min. The 4-OHT/corn oil solution was then loaded into insulin syringes (BD; catalog #329461) at a dose of 50 mg/kg for injection. Clozapine-n-oxide (CNO; Tocris; catalog #4936) was dissolved in 0.5% DMSO (Sigma; catalog #D2438; v/v in 0.9% sterile saline) at room temperature to a concentration of 3.79 mg/ml. The solution was then aliquoted and stored at −20°C until experimentation. A dose of 2.5 mg/kg was used for injection; 0.5% DMSO (v/v in 0.9% sterile saline) was used as a vehicle control injection.

### Viral injections for DREADD experiments

pAAV-hSyn-DIO-hM4D(Gi)-mCherry (Addgene; catalog #44362-AAV9; RRID:Addgene_44362) or pAAV-hSyn-DIO-mCherry (Addgene; catalog #50459-AAV9; RRID:Addgene_50459) was used for bilateral intracranial injections into the AOB of resident ArcTRAP mice (Krashes et al., 2011). Mice were head-fixed on a custom stereotaxic device that allows for a 30° tilt along the horizontal plane, elevating the rostral brain. A Nanoject II (Drummond Instruments) was used to deliver a volume of 92-110 nl at coordinates AP 3.80 mm, ML ±1.00 mm, and DV −2.00 mm relative to bregma. After 1 week of recovery, mice were TRAPed. The mice were then given another week to allow for expression of the Cre-dependent G_i_-DREADD. Subsequently, the mice were exposed to repeated resident intruder interactions following intraperitoneal injections with either DMSO (control; 0.5% DMSO v/v in 0.9% sterile saline) or CNO (2.5 mg/kg in 0.5% DMSO).

### Tissue imaging

Fixed tissue imaging was conducted at the URMC Histology, Biochemistry, and Molecular Imaging core facility using an Olympus VS120 Virtual Slide Microscope and Visiopharm Image Analysis System. Resident mice were TRAPed on day 0 where they received either 4-OHT and the resident–intruder assay, 4-OHT alone, or the resident–intruder assay alone. On day 3, mice were euthanized with ketamine (300 mg/kg)/xylazine (30 mg/kg) and intracardially perfused with 0.9% sodium chloride, followed by 4% PFA. Brains were dissected and postfixed in 4% PFA for 24-48 h; 30% sucrose was used to cryoprotect the brains overnight before embedding in OCT compound (Fisher) for cryosectioning on a Shandon Cryotome FSE (Thermo Electron). Sections were cut at 25 µm thickness and free-floated in PBS before mounting. Sections were mounted using Vectashield Hardset antifade mounting medium (Vector Laboratories); 20× *Z*-stack (15 slices, 1.34 μm spacing) images were captured in the TRITC channel (580 nm emission wavelength).

Live tissue 2-photon imaging was conducted on ArcTRAP-d4EGFP mice. Images were captured on a Thorlabs Acerra upright 2-photon microscope system equipped with an XLUMPlanFLN 20× objective (Olympus) and a fast-scanning resonant galvanometer along one of the two principal axes. ArcTRAP-d4EGFP residents were TRAPed on day 0. On days 1 and 2, they were re-exposed to the intruder seen on day 0. On day 3, residents were either re-exposed to the same intruder seen previously or exposed to a novel female. Three hours after the day 3 resident–intruder interaction, brains were acutely dissected and live slices were prepared (as described above); 200-µm-thick image stacks were taken from the AOB with ∼0.5 × 0.5 × 2 µm sampling, with excitation at 890 nm and emission filters for eGFP (525 ± 50 nm) and tdTomato (607 ± 70 nm). Each image was generated by averaging 16 sequentially acquired frames.

### Experimental design and statistical analysis

For fixed tissue image analysis, images were first opened in QuPath (Bankhead et al., 2017) and converted to .tiff format for processing in FIJI/ImageJ (Schindelin et al., 2012). Maximum intensity projections were used, and brightness/contrast was adjusted equally for all images. A threshold was set to include tdTomato^+^ cell bodies, and the watershed algorithm was applied to separate cell bodies in close proximity. An ROI was drawn around the ICL of the AOB. Particles were included based on size and circularity, and analyzed by the built-in “analyze particles” ImageJ function. Cell counts were normalized to the ROI area. Results were analyzed via repeated-measures one-way ANOVA using Geisser–Greenhouse correction. Comparisons between individual treatments were made using Holm–Šídák multiple comparisons tests.

For live 2-photon image analysis, images were stitched in 3 dimensions using ImageJ (Schindelin et al., 2012). From the stitched 3D images, we selected 20 continuous frames (40 µm) in depth containing the ICL for analysis. Background subtraction and intensity normalization were applied to each image stack. An ROI was drawn around the ICL, and colocalization analysis was performed on 3D volumes using the Colocalization Threshold plugin for ImageJ. Thresholded Manders coefficients were calculated for both tdTomato and eGFP channels and reported (Manders et al., 1993). Each AOB was roughly bisected along its medial-lateral axis, and AOBs from both hemispheres were imaged. This resulted in 4 imaged sections from each animal. Mixed-effects ANOVA analysis was performed using Geisser–Greenhouse's correction (no assumption of sphericity).

Multidimensional analysis was performed across all IGCs recorded in both the single exposure (SE) and RE conditions (see Fig. 5). Clustering was performed for 137 cells across 23 features using the Density Based Merging approach (Walther et al., 2009) embedded within the uniform manifold approximation (McInnes et al., 2018) package for MATLAB. We set the DBM “cluster detail” option to “very low” (i.e., setting a very high threshold for splitting clusters into subclusters, resulting in a small/conservative number of clusters). Inspecting members of each cluster (see, i.e., Fig. 5*B*) allowed us to identify Cluster 1 as containing IGCs with a low-excitability phenotype, and Cluster 3 as containing IGCs with a high-excitability phenotype. Linear discriminant analysis (LDA) was used to identify an Eigenvector that maximally separates Cluster 1 and Cluster 3 populations (see Fig. 5*C*). By projecting all cells (including those in Cluster 2) along this Eigenvector, we were able to quantify the proximity of cells to the high-excitability (Cluster 3) or low-excitability (Cluster 1) phenotype.

For analysis of CNO effects on IGC resting potential (see Fig. 7*C*), average membrane potential during the 0-2, 10-12, and 30-32 min windows of gap-free recordings was calculated and compared. The DREADD+CNO condition comprised 17 recordings from 13 animals, and the mCherry+CNO condition comprised 6 recordings from 3 animals. Mixed-effects ANOVA tests were performed across the measurement windows with Šídák correction for multiple comparisons.

Aggressive behaviors were quantified by manual video review with the assistance of a custom MATLAB interface that blinds the scorer to the experimental conditions and DeepLabCut, a computer software package used for animal pose estimation based on transfer learning with deep neural networks (Mathis and Mathis, 2020). DeepLabCut was trained with 1184 video frames that were manually annotated, and 12 body labels were applied to the resident and intruder. From the manual annotation, we identified 20 resident behaviors that could be analyzed for state duration (occupancy). Overall, 259 behavioral features/metrics were calculated based on the tracking data generated from DeepLabCut, including the following: distance, velocity, body orientation, and position. A random forest classifier was applied to classify the 20 behaviors of interest. After training, the performance of the random forest classifier was evaluated by fivefold cross-validation, and the accuracy of the final classifier was 0.943. For manual video analysis, the number of resident-initiated aggressive interactions were counted. These aggressive interactions included attack behavior (lunging at the intruder with or without biting) and fighting behavior (active physical altercation between the resident and intruder, often involving tumbling). Sequential behaviors were counted separately if a physical separation occurred between the resident and intruder, or if there was at least a 2 s pause in the interaction.

## Results

### *Arc* expression is upregulated in AOB IGCs after the resident–intruder paradigm

**Figure 1.**
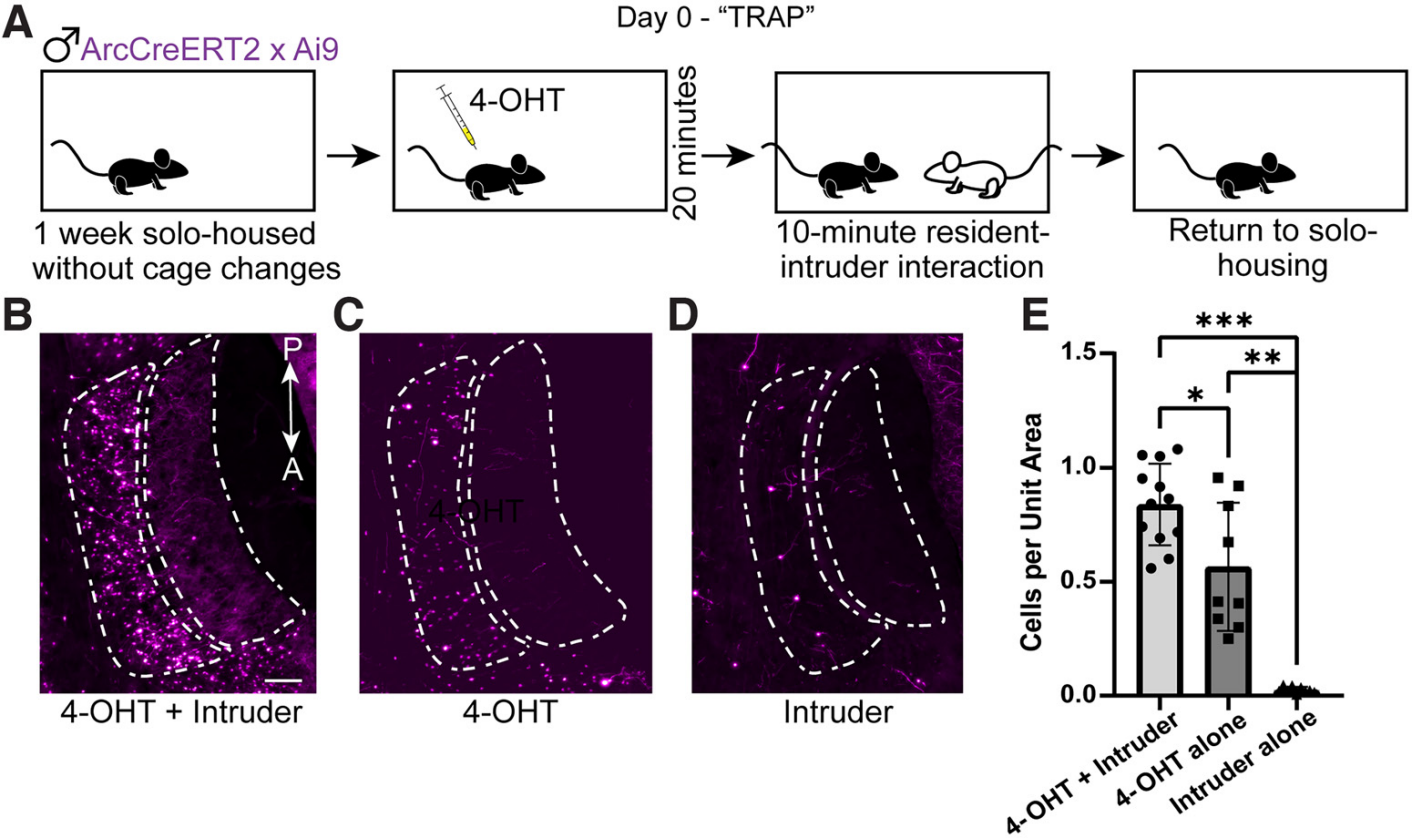
TRAPing during resident–intruder interactions permanently labels AOB IGCs with tdTomato. ***A***, The TRAPing process (paired 4-OHT injection and social interaction). Day 0 is TRAP Day. ***B–D***, Sample AOB images of tdTomato expression in ArcTRAP mice in 4-OHT+Intruder (***B***), 4-OHT alone (***C***), and Intruder alone (***D***) conditions. Animals were perfused 7-14 d after the TRAPing process. In all three images, the ICL is outlined by the dashed region to the left, and the external cellular layer and glomerular layer are outlined by the dashed region to the right. tdTomato^+^ cells in the ECL are EGCs, another AOB interneuron type. Scale bar, 100 µm. ***E***, Quantification of tdTomato-positive cells in the ICL. Cell counts are normalized to the ICL area. Error bars indicate mean ± SD. Repeated-measures one-way ANOVA, *F*_(1.002,2.003)_ = 98.73, *p* = 0.0099. Holm–Šídák correction for multiple comparisons. *p = 0.04. **p = 0.0196. ***p = 0.0063. *n* = 9-12 AOB sections from 3 or 4 mice per treatment.

To study *Arc*-expressing IGCs over week-long time periods, we first wanted to validate the use of the ArcTRAP transgenic mouse line. This transgenic mouse contains a tamoxifen-inducible Cre (CreERT2) directed by the *Arc* promoter. When *Arc* expression occurs, it causes expression of tamoxifen-inducible Cre. If tamoxifen is present, Cre will excise the stop codon on the Ai9 transgene, permanently labeling that neuron with tdTomato. We tested whether tdTomato expression was elevated in mice after inducing CreERT2 with 4-OHT and subjecting male mice to the resident–intruder assay, which has been used to model male–male territorial aggression. Resident male ArcTRAP mice were solo-housed for 1 week before behavioral testing. On day 0, resident mice received an injection of 4-OHT and were left in their home cage to habituate for 20 min. Following the 20 min habituation period, a male BALB/cJ intruder mouse was introduced to the resident's home cage for 10 min (Fig. 1*A*). This combination of solo-housing and 4-OHT injection+behavior on day 0 will be referred to as TRAP throughout the text. Residents that received both the 4-OHT injection and the resident–intruder assay showed a significant increase in tdTomato expression in the AOB ICL 7–14 d after the injection, compared with residents that received either the 4-OHT injection in their home cages or resident–intruder assay without 4-OHT injections (Fig. 1*B–E*). The small number of labeled IGCs in the absence of 4-OHT injections (CreERT2 “leak”) is a concern in some brain regions of ArcTRAP mice (Guenthner et al., 2013) but is minimal in the AOB.

We observed a small number of tdTomato^+^ neurons in the AOB ECL, where excitatory mitral cells and inhibitory external granule cells (EGCs) reside. The labeled neurons were almost certainly EGCs, as they (1) have a small (<10 µm) spherical soma, (2) highly branched, radially symmetric, spine-dense dendrites, (3) a lack of visible dendritic tufts in the glomerular layer, and (4) no visible axon (Larriva-Sahd, 2008; Maksimova et al., 2019; Zhang and Meeks, 2020). The presence of a small number of ArcTRAP^+^ EGCs suggests that these cells may also undergo experience-dependent plasticity, which warrants future investigation. Because *Arc* is induced in AOB IGCs through the detection of accessory olfactory chemosignals in the mouse's environment (Cansler et al., 2017), we were not surprised to see some tdTomato expression in those residents only receiving the 4-OHT injection. This indicates that the residents are able to detect their own chemosignals in their home cage, and that this detection is enough to induce some level of *Arc* expression in select IGCs, or that there is a baseline expression of *Arc* in IGCs. Overall, these results support the use of the ArcTRAP transgenic mouse line for measuring and tracking *Arc*-expressing IGCs across time in the context of male–male territorial aggression.

### ArcTRAP^+^ IGCs show increased intrinsic excitability for days after a single resident–intruder interaction

Previous studies indicated that *Arc*-expressing IGCs display changes in their intrinsic excitability 4-8 h after a single resident–intruder assay (Cansler et al., 2017) or mating (Gao et al., 2017). ArcTRAP^+/−^ × Ai9 mice (Fig. 1) allow for permanent labeling of *Arc*-expressing cells, but these animals are also hemizygous for *Arc*, which was not the case in Arc-d4EGFP mice studied in Cansler et al. (2017), the latter being generated via bacterial artificial chromosome transgenesis (Grinevich et al., 2009). Here, we sought to determine whether *Arc*-hemizygous AOB IGCs also increase their excitability following resident–intruder interactions, and, if so, to determine the length of intrinsic excitability changes. We hypothesized that, if *Arc*-expressing IGCs were playing a role in regulating behaviorally relevant incoming chemosensory information, we would see some persistent changes in excitability across a longer time period.

**Figure 2.**
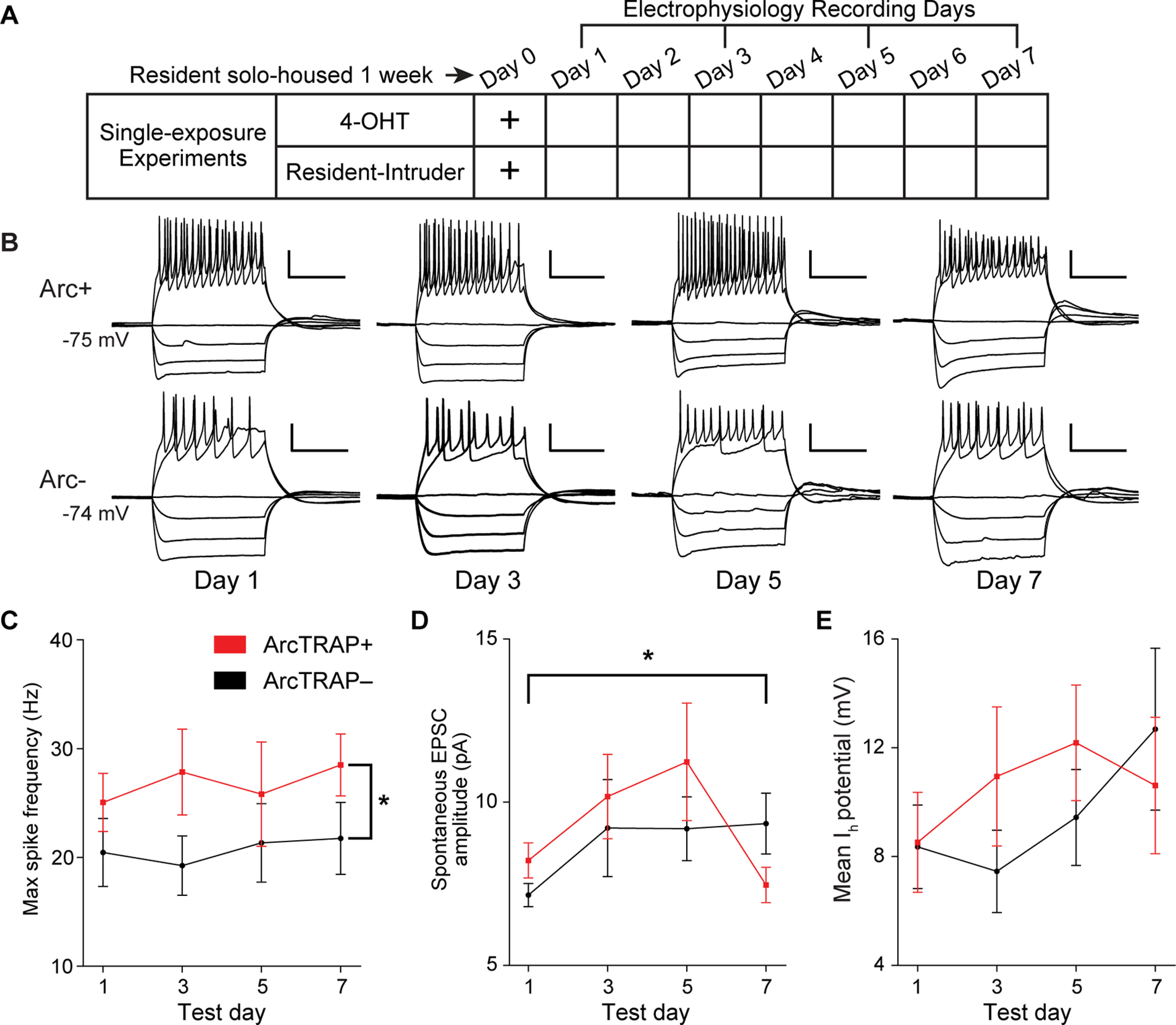
ArcTRAP^+^ IGCs show increased intrinsic excitability for 1-3 d following a single resident–intruder interaction. ***A***, Depiction of resident–intruder interaction followed by electrophysiology recordings on day 1, 3, 5, or 7. ***B***, Sample current-clamp traces from ArcTRAP^+^ and ArcTRAP^–^ IGCs on days 1, 3, 5, and 7. Calibration: 0.25 s, 25 mV. ***C***, Maximum spiking frequency of Arc^+^ and Arc^–^ IGCs across days. Error bars indicate mean ± SEM. *Mixed-effects ANOVA, fixed effect of fluorescence, *F*_(1,63)_ = 5.9266, *p* = 0.018. ***D***, Spontaneous EPSC amplitude of ArcTRAP^+^ and ArcTRAP^–^ IGCs across days. Error bars indicate mean ± SEM. *Mixed-effects ANOVA, fixed effect of test day, *F*_(3,63)_ = 2.8039, *p* = 0.047. ***E***, Mean I_H_ sag potential of ArcTRAP^+^ and ArcTRAP^–^ IGCs across days. Error bars indicate mean ± SEM. Mixed-effects ANOVA, not significant. ***C-E***, *n* = 33 ArcTRAP^+^ cells, 38 ArcTRAP^–^ cells, 16 animals.

Resident mice were TRAPed on day 0 via a resident–intruder interaction with a BALB/cJ male. Following this single resident–intruder assay, acute slices were prepared for whole-cell patch-clamp electrophysiology on day 1, 3, 5, or 7. We recorded a series of electrophysiological challenges in current clamp (I-Clamp) and voltage clamp (V-Clamp) from tdTomato-positive (ArcTRAP^+^) and tdTomato-negative (ArcTRAP^–^) IGCs. Sample I-Clamp traces from ArcTRAP^+^ and ArcTRAP^–^ IGCs on day 1, 3, 5, and 7 can be seen in Figure 2*B*. We measured 23 electrophysiological features from I-Clamp and V-Clamp challenges, which we have previously used to assist in identifying differing qualities within and across AOB interneuron types (Maksimova et al., 2019). Previous results obtained during a 4-8 h time window following social behavior indicated that maximal spiking frequency increased in *Arc*-expressing IGCs (Cansler et al., 2017). We therefore investigated whether ArcTRAP^+^ and ArcTRAP^–^ IGCs also demonstrated differences in spiking frequency (Fig. 2*C*). We found that ArcTRAP^+^ IGCs had higher maximum spiking frequencies than the ArcTRAP^–^ population (mixed-effects ANOVA, fixed effect of fluorescence, *F*_(1,66)_ = 5.85, *p* = 0.018, Fig. 2*C*). We investigated other electrophysiological features across fluorescence type and test day (Fig. 2*C–E*). We observed a trend toward a time-dependent difference in spontaneous EPSC amplitudes, but no significant effect between ArcTRAP^+^ and ArcTRAP^–^ groups (mixed-effects ANOVA, EPSC fixed effect of test day *F*_(3,66)_ = 2.64, *p* = 0.057; fixed effect of fluorescence *F*_(1,66)_ = 0.864, *p* = 0.356; Fig. 2*D*). We did not see a difference in the hyperpolarization-activated (I_H_) sag potentials, a notable feature of all IGCs (Fig. 2*E*) (Hu et al., 2016). These data indicated that differences in ArcTRAP^+^ IGCs persisted across a long, behaviorally relevant time scale.

### A similar population of IGCs re-express *Arc* on RE to the same intruder mouse

**Figure 3.**
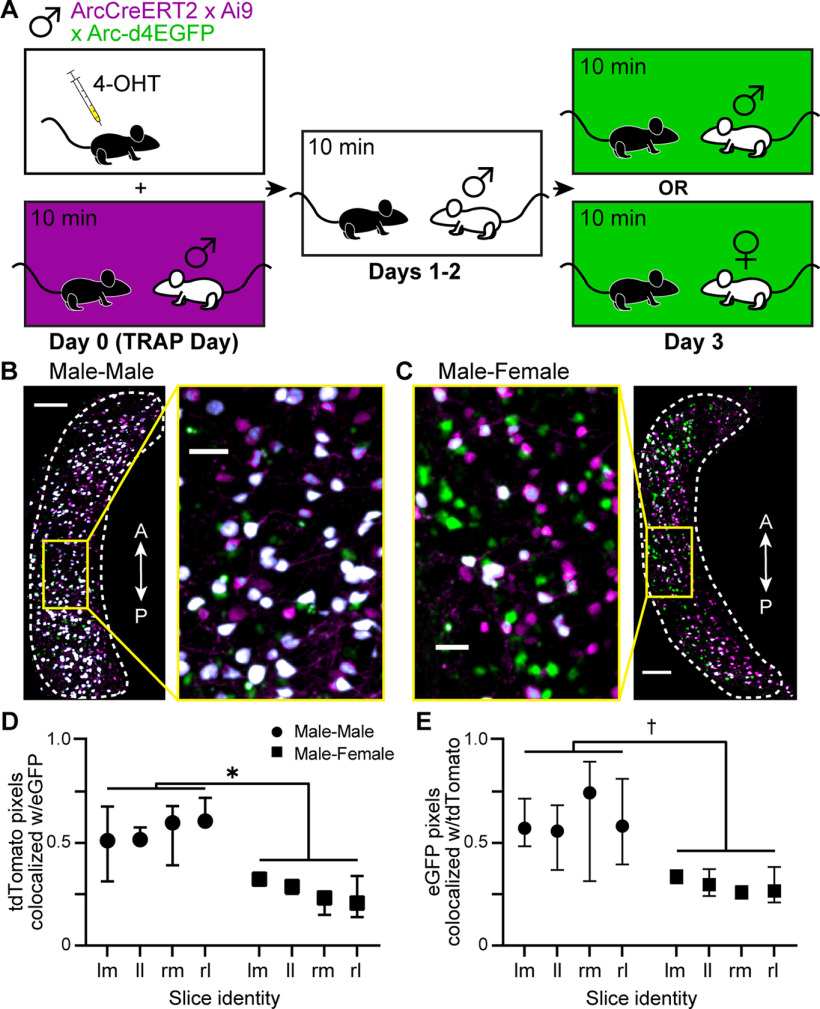
RE to the same intruder induces *Arc* expression in a similar population of IGCs across multiple days. ***A***, Depiction of repeated resident–intruder behavioral paradigm involving triple transgenic (ArcTRAP × Ai9 × Arc-d4eGFP) mice. 4-OHT was injected 20 min before resident–intruder exposure on day 0. On day 3, resident mice were either exposed to the same male intruder seen on days 0-2 or to a novel female intruder. ***B***, Left, AOB image from a resident exposed to the same male intruder on all 4 d. Scale bar, 100 µm. Right, ICL inset from male–male exposure. Scale bar, 25 µm. Magenta represents tdTomato expression driven by ArcTRAP procedure on day 0. Green represents eGFP induced by *Arc* expression on day 3. White represents colocalization between tdTomato and eGFP. Dashed line outlines the ICL. A, Anterior; P, posterior. ***C***, Right, AOB image from a resident exposed to the same male intruder for 3 d, and a novel female intruder on the fourth day. Scale bar, 100 µm. Left, ICL inset from male–female exposure. Scale bar, 25 µm. ***D***, Colocalization (thresholded Manders coefficient) of tdTomato^+^ pixels (magenta) with eGFP pixels (green). *Mixed-effects ANOVA, fixed effect of treatment, *F*_(1,4)_ = 27.81, *p* = 0.0062. Four sections per animal, 3 animals. ***E***, Colocalization of eGFP^+^ pixels with tdTomato pixels. †Mixed-effects ANOVA, fixed effect of treatment, *F*_(1,4)_ = 6.806, *p* = 0.0595. Four sections per animal, 3 animals. lm, Left hemisphere/medial AOB; ll, left hemisphere/lateral AOB; rm, right hemisphere/medial AOB; rl, right hemisphere/lateral AOB.

Given their days-long increases in excitability, we hypothesized that *Arc*-expressing AOB IGCs may contribute to experience-dependent chemosensory learning. For example, *Arc*-expressing IGCs may inhibit the population of AOB mitral cells activated during a salient social behavior, like aggression, and continue shaping mitral cell activation through repeated interactions. If *Arc*-expressing IGCs were indeed contributing to social behavior plasticity, we hypothesized that similar populations of IGCs would become activated during repeated behavior interactions with the same mouse. To test this hypothesis, we used triple-transgenic ArcTRAP-d4EGFP mice. In addition to the tamoxifen-inducible Cre and tdTomato reporter (ArcTRAP), these mice harbor a bacterial artificial chromosome that contains a transiently active EGFP induced by the *Arc* promoter (Arc-d4EGFP) (Grinevich et al., 2009). These triple-transgenic mice allow us to permanently label cells expressing *Arc* on day 0 with tdTomato, and then transiently label *Arc*-expressing cells on subsequent behavioral testing days with d4EGFP (Fig. 3*A*). Male resident ArcTRAP-d4EGFP mice were TRAPed on day 0 with a male BALB/cJ intruder. On days 1 and 2, the resident was re-exposed to the same BALB/cJ intruder in the resident–intruder assay. On day 3, resident mice were either exposed to the same BALB/cJ intruder seen on the previous days or introduced to a female BALB/cJ. Three hours after the final resident–intruder assay on day 3, acute slices were prepared for 2-photon imaging. Colocalization analysis was performed to determine how much overlap existed between the tdTomato and d4EGFP signals in male–male and male–female exposed mice (Fig. 3*B*,*C*). Residents that were exposed to the same male mouse on all 4 d of the testing had increased colocalization of tdTomato and d4EGFP (Fig. 3*D*,*E*) compared with residents that were exposed to a male on the first 3 d and a female on the last day. These data show that IGCs re-express *Arc* after multiple social experiences with the same mouse, and that the patterns of ArcTRAP IGC labeling after by male and female interactions are distinct.

### ArcTRAP^+^ IGCs sustain increased intrinsic excitability across daily resident–intruder interactions

**Figure 4.**
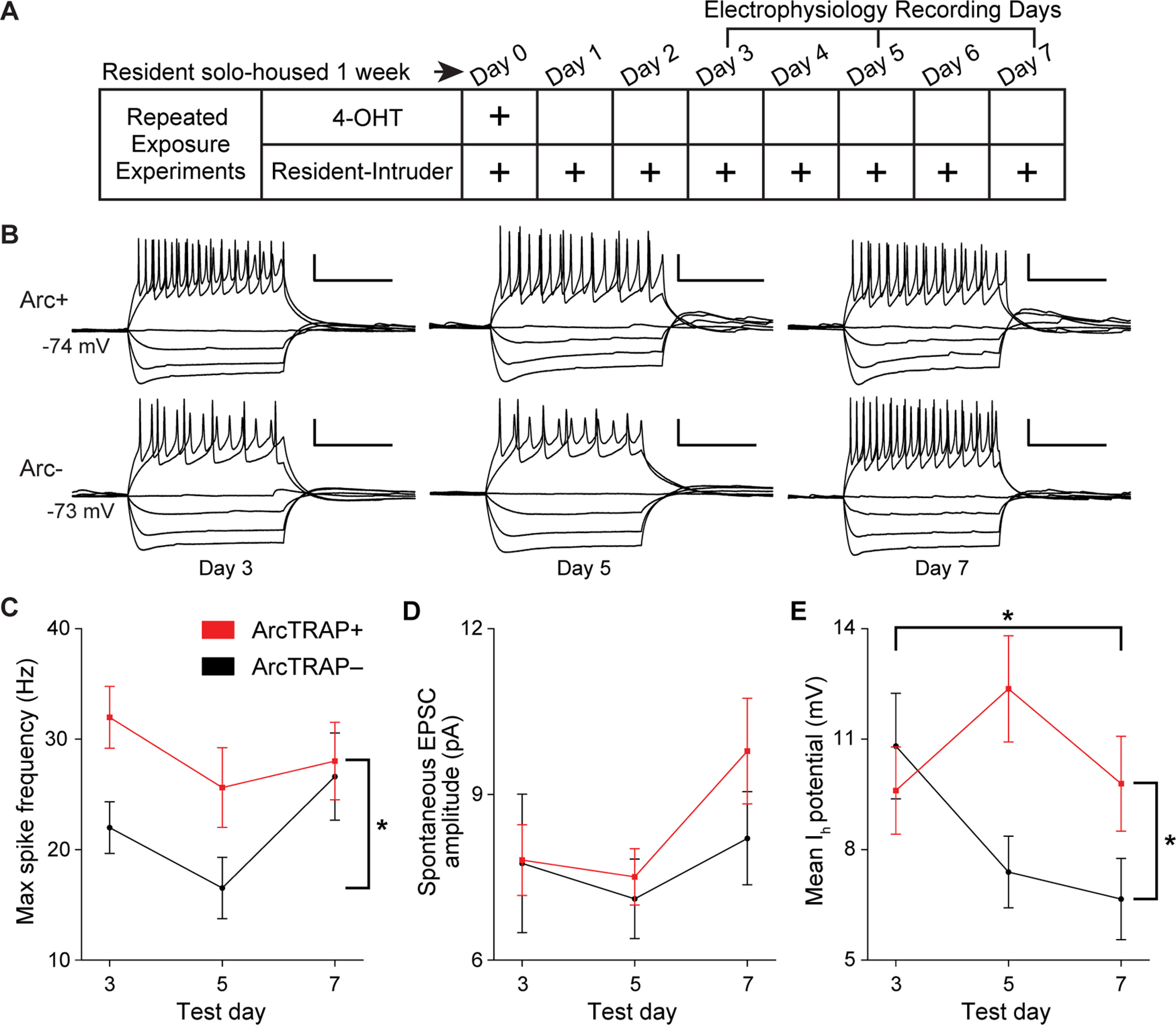
ArcTRAP^+^ IGCs show increased intrinsic excitability after repeated resident–intruder interactions. ***A***, Resident–intruder interactions followed by electrophysiology recordings on day 3, 5, or 7. ***B***, Sample current-clamp traces from ArcTRAP^+^ and ArcTRAP^–^ IGCs on days 3, 5, and 7. Calibration: 0.25 s, 25 mV. ***C***, Maximum spiking frequency of ArcTRAP^+^ and ArcTRAP^–^ IGCs across days. Error bars indicate mean ± SEM. *Mixed-effects ANOVA, fixed effect of fluorescence, *F*_(1,61)_ = 6.4302, *p* = 0.014. ***D***, Spontaneous EPSC amplitude of ArcTRAP^+^ and ArcTRAP^–^ IGCs across days. Error bars indicate mean ± SEM. Mixed-effects ANOVA, not significant. ***E***, Mean I_H_ sag potential of ArcTRAP^+^ and ArcTRAP^–^ IGCs across days. Error bars indicate mean ± SEM. *Mixed-effects ANOVA, fixed effect of fluorescence, *F*_(1,61)_ = 5.267, *p* = 0.025; interaction between test day and fluorescence, *F*_(2,61)_ = 3.2199, *p* = 0.047. ***C-E***, *n* = 31 ArcTRAP^+^ cells, *n* = 36 ArcTRAP^–^ cells, 17 mice.

Having shown that ArcTRAP^+^ IGC excitability is elevated for days following a single resident–intruder interaction, and that ArcTRAP^+^ IGCs are reactivated by repeated interactions with the same animal, we proceeded to investigate the physiological responses of ArcTRAP^+^ IGCs during repeated resident–intruder interactions. If ArcTRAP^+^ IGCs show increased excitability during repeated social encounters, this would further suggest that these cells are capable of supporting AOS-mediated social behavior plasticity. To test this, we conducted similar electrophysiology experiments to those described in Figure 2. However, in this cohort, we re-exposed resident mice to the same intruder every day leading up to the day of electrophysiology recording, including the day of recording (Fig. 4*A*). On day 3, 5, or 7, acute slices were prepared for whole-cell patch-clamp electrophysiology. The same I-Clamp and V-Clamp challenges were applied to ArcTRAP^+^ and ArcTRAP^–^ IGCs (Fig. 4*B*). We found that ArcTRAP^+^ IGCs had a significantly increased maximum spiking frequency compared with ArcTRAP^–^ IGCs (Fig. 4*C*). We did not see a significant difference in spontaneous EPSC amplitudes when the mice underwent repeated resident–intruder exposures (Fig. 4*D*). Significant increases in I_H_ currents were found with RE in the ArcTRAP^+^ population (Fig. 4*E*), which were not seen with SE. These results indicate that ArcTRAP^+^ IGCs maintain increased intrinsic excitability features across multiple behavior exposures, suggesting a role in AOS-mediated behavioral plasticity.

**Figure 5.**
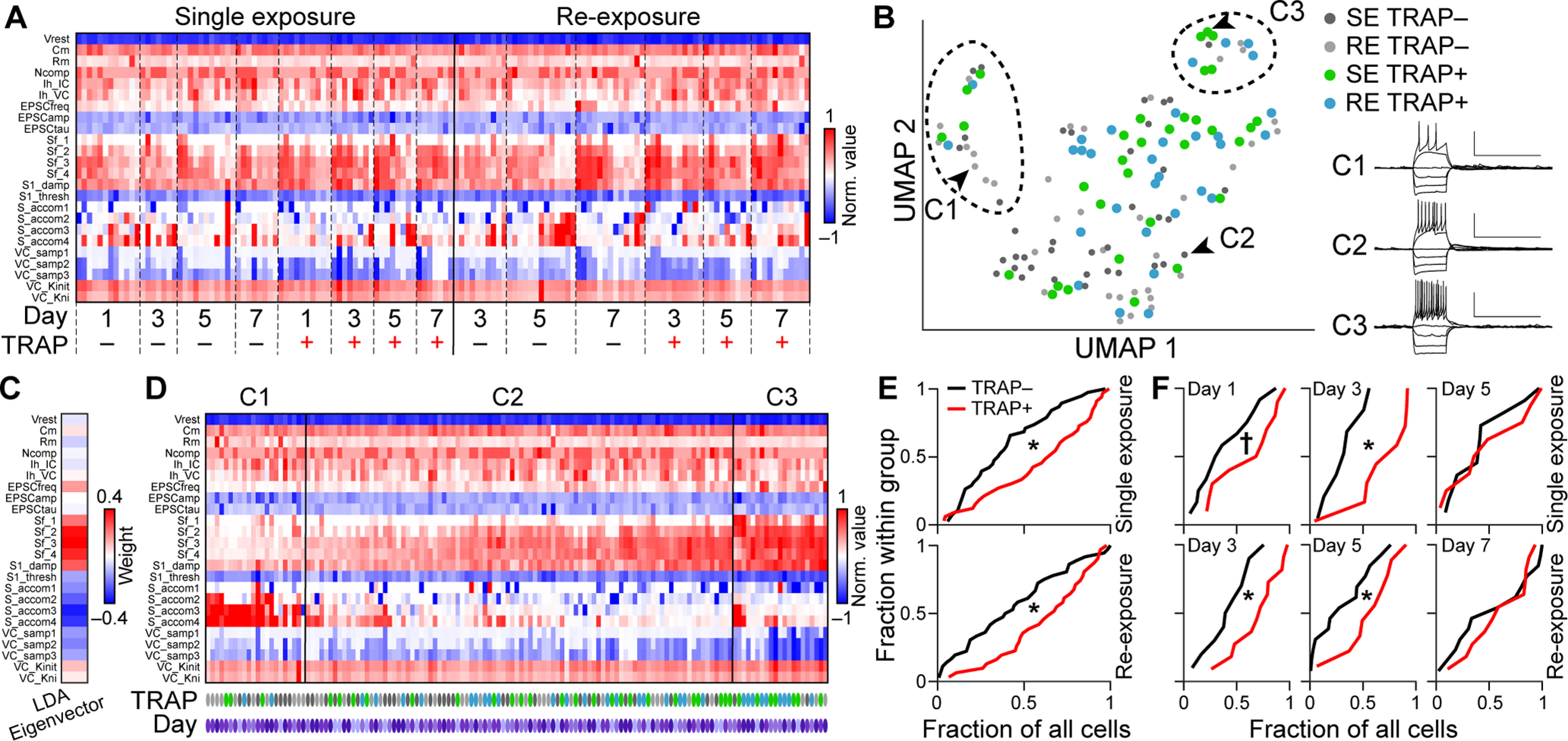
Multidimensional analysis of IGC electrophysiological properties. ***A***, Heatmap of 23 electrophysiological properties recorded for 137 IGCs included in SE and RE experiments (Fig. 2 and Fig. 4, respectively). Measurements were normalized by the maximum value observed and colorized based on the sign of the measurement for display purposes. ***B***, Uniform manifold approximation and projection (UMAP) of all cells in ***A***, colorized by their identity. Despite high levels of heterogeneity, cluster analysis identified IGCs with low excitability (***C1***), high excitability (***C3***), and intermediate excitability (***C2***). Insets, Cells identified by arrowheads. Calibration: 50 mV, 1 s. ***C***, Heat map display of (***C1-C3***) LDA Eigenvector weights, used for projections in ***D-F***. ***D***, Clustered heat map with cells displayed based on their projection along an Eigenvector that best separates cells in Cluster 1 (low excitability) from cells in Cluster 3 (high excitability). Purple ovals represent day of measurement (lightest, day 1; darkest, day 7), and TRAP condition is indicated using the same color codes as in ***B***. ***E***, Cumulative distributions of TRAP^+^ (red) and TRAP^–^ (black) IGCs along the Eigenvector indicated in ***C*** for SE (top) and RE (bottom) experiments. ***F***, Relative ranks of TRAP^+^ and TRAP^–^ IGCs along LDA Eigenvector, broken down by experimental day. *p < 0.05; †*p* < 0.1; Kruskal–Wallis test.

The changes in ArcTRAP^+^ IGC physiology we saw in both single and repeated resident–intruder interactions were largely consistent with those observed in d4EGFP-expressing cells 4-8 h after resident–intruder interactions (Cansler et al., 2017). Previous work indicated that IGCs are electrophysiologically heterogeneous (Cansler et al., 2017; Gao et al., 2017; Maksimova et al., 2019) but that they can be readily distinguished from other AOB IGCs based on multidimensional analysis of electrophysiological parameters (Maksimova et al., 2019). In order to more systematically investigate the impacts of these behavioral conditions on ArcTRAP^+^ and ArcTRAP^–^ IGCs, we analyzed 137 IGCs recorded in both SE and repeated exposure (RE) conditions (Fig. 5). Cluster analysis identified both a “high-excitability” and a “low-excitability” IGC population across all recordings (Fig. 5*B*). The low-excitability cluster (Cluster 1) was enriched for ArcTRAP^–^ IGCs across days and experiments (17 of 22 cells, *p* < 0.01, binomial test), whereas the high-excitability cluster (Cluster 3) was enriched for ArcTRAP^+^ IGCs (16 of 21 cells, *p* < 0.01, binomial test). The majority of IGCs were not included in either of these clusters (94 of 137, *p* = 0.54, binomial test), suggesting that excitability changes are not all-or-none but are instead multidimensional. In order to determine whether ArcTRAP^+^ cells are systematically shifted toward the high-excitability state, we used LDA to generate a multidimensional vector (Eigenvector) that best distinguished high-excitability and low-excitability clusters (Fig. 5*C*). Projecting each recording along this primary LDA Eigenvector allowed us to quantify the relative closeness of each IGC to the high-excitability or low-excitability state (Fig. 5*D*,*E*). This comparison revealed strong separation of ArcTRAP^+^ and ArcTRAP^–^ IGCs in both SE and RE conditions (Fig. 5*D*). To facilitate statistical comparisons, we ranked all cells on an axis from 0 to 1, with 0 being the low-excitability extreme and 1 being high-excitability extreme (Fig. 5*E*,*F*). We then compared the ranks of all ArcTRAP^+^ and ArcTRAP^–^ cells in both SE and RE conditions, finding in both cases that ArcTRAP^+^ cells were shifted toward the high-excitability end compared with ArcTRAP^–^ cells (Fig. 5*E*). For the sake of visualization, the vertical axes in Figure 5*E*, *F* are normalized by the total number of cells applicable in the comparison (“Fraction within group”). Furthermore, this comparison confirmed that ArcTRAP^+^ IGC excitability increases were present at 1 and 3 d after TRAPing in SE experiments, and that these increases were present at 3 and 5 d after TRAPing in RE experiments (Fig. 5*E*). In day 7 RE experiments, we observed no difference in ArcTRAP^+^ and ArcTRAP^–^ excitability (*p* = 0.71, Kruskal–Wallis test). This appeared to be because of an increase in ArcTRAP^–^ excitability, rather than a decrease in ArcTRAP^+^ excitability (Fig. 5*C*). Given that each resident–intruder interaction activates some IGCs that were not activated on “TRAP Day” (Fig. 3), this lack of separation on day 7 may reflect the inclusion of cells that began expressing *Arc* on days 1-7 (after TRAP), and therefore did not express tdTomato at the time of recording. Overall, these results confirm and extend the observations that *Arc*-expressing AOB IGCs shift toward high-excitability states for days following resident–intruder interactions, in both SE and RE conditions.

### Aggressive behavior in male mice increases across repeated resident–intruder interactions

**Figure 6.**
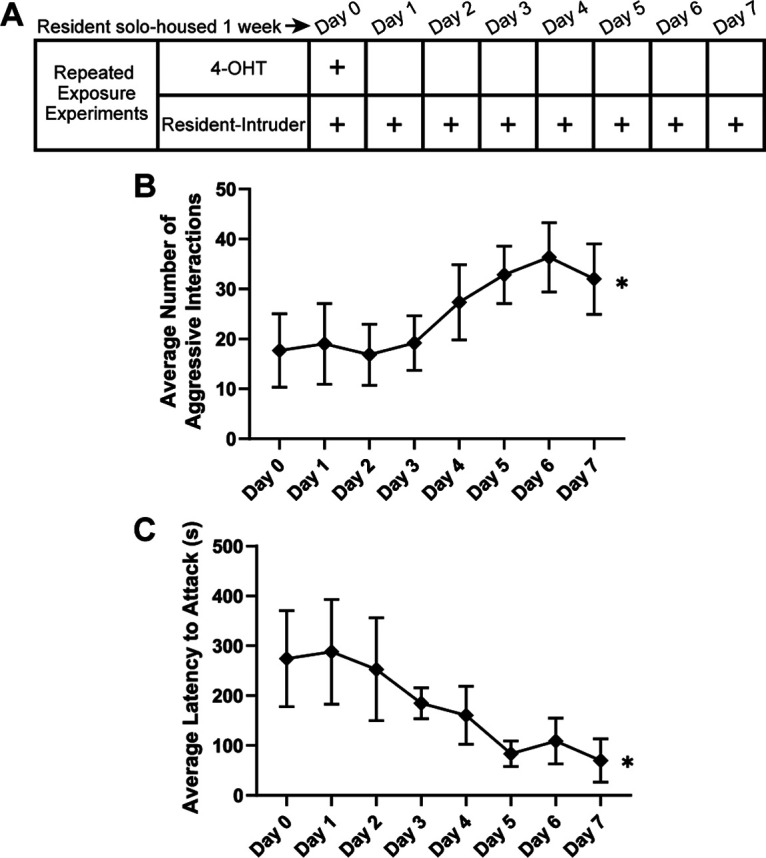
Ramping aggression in ArcTRAP males during repeated resident–intruder interactions. ***A***, Depiction of resident–intruder schedule across 8 d. ***B***, Average number of resident-initiated aggressive interactions across repeated resident–intruder interactions. *Repeated-measures ANOVA, *F*_(7,35)_ = 5.041, *p* = 0.0005, *n* = 6. ***C***, Average latency to first attack across repeated resident–intruder interactions. *Repeated-measures ANOVA, *F*_(7,35)_ = 3.579, *p* = 0.0052. Error bars indicate mean ± SEM.

When conducting the repeated exposures to the resident–intruder assay, we noted changes in the resident's aggression levels over time. Given that intermale aggression is AOS-mediated (Stowers et al., 2002), we investigated whether this aggression is supported by experience-dependent plasticity in the AOB. Other studies have indicated that male resident mice tend to become more aggressive when undergoing short-duration, repeated resident–intruder interactions (Sunkin et al., 2013). Because of this, we wanted to identify whether male ArcTRAP resident mice increase their aggressive behavior across repeated, 10 min resident–intruder interactions once per day for 8 d. Video was recorded during the repeated resident–intruder interactions for electrophysiology. We manually quantified the behavior by counting the number of resident-initiated aggressive interactions, as well as the latency to the first aggressive interaction (Fig. 6*B*,*C*). We found that resident mice significantly increased the number of aggressive interactions toward the end of the 8 d behavior period (days 5-7) (Fig. 6*B*). In line with an increase in aggression, we also saw a decrease in the latency to the first attack, with a significant decrease in latency between day 3 and day 5 of testing (Fig. 6*C*). These results indicate that experimental conditions that reactivate ArcTRAP^+^ AOB IGCs coincide with changes in an AOS-mediated social behavior.

### Inhibiting ArcTRAP^+^ IGCs with DREADDs abolishes the intermale ramping aggression

**Figure 7.**
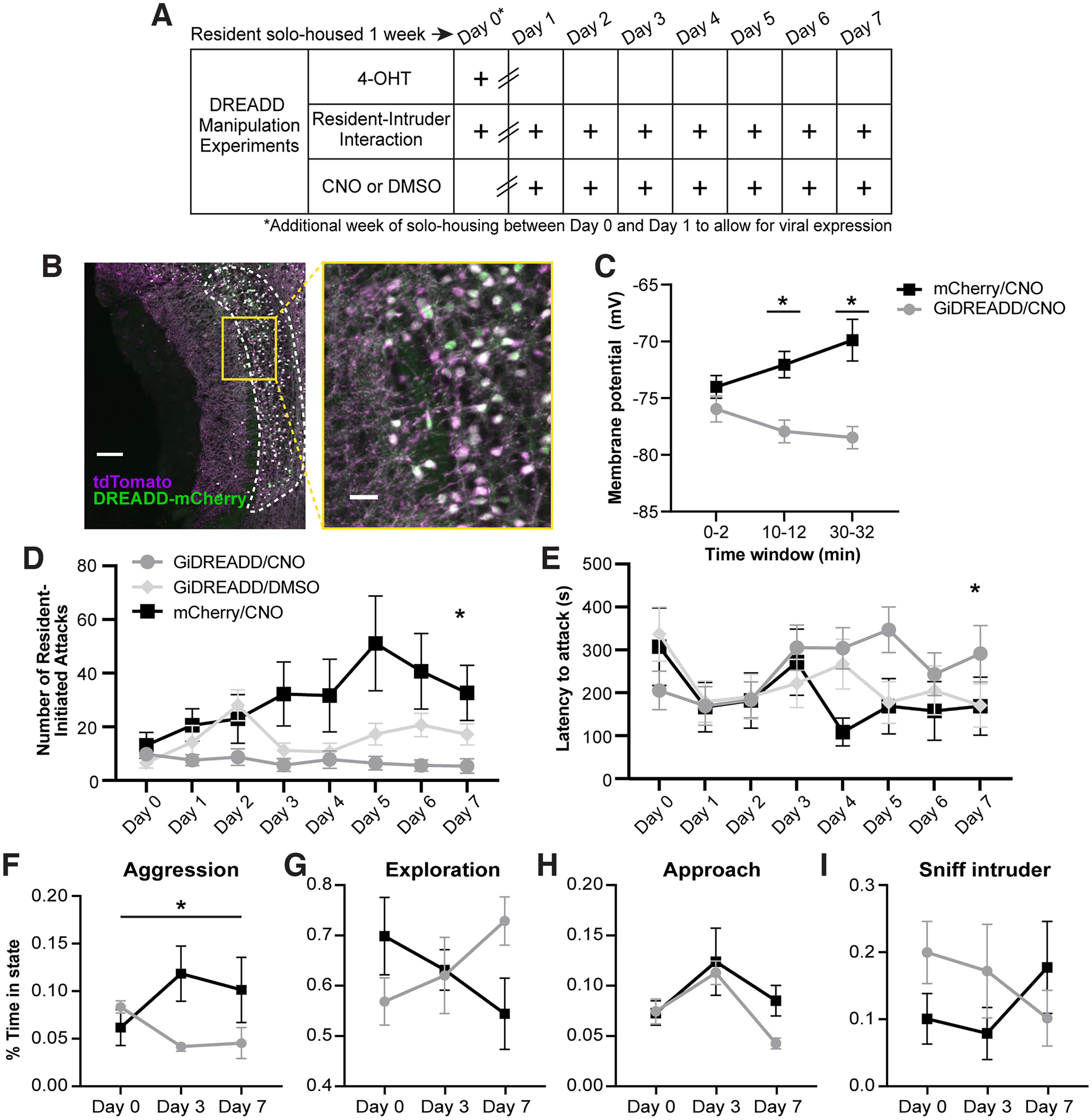
Inhibiting ArcTRAP^+^ IGCs with a Gi-DREADD abolishes ramping aggression in male mice. (***A***) Depiction of the resident–intruder interaction schedule with drug injections. Mice were solo-housed for 1 week before day 0, and for an additional week after day 0. Subsequently, they received daily CNO or DMSO (control) injections before the resident–intruder paradigm for the duration of the experiment. (***B***) AAV-GiDREADD-mCherry colocalization with ArcTRAP^+^ (tdTomato^+^) IGCs. Scale bars: 100 µm (left), 25 µm (right). (***C***) Whole-cell patch-clamp membrane potential recordings from IGCs in AOB slices during CNO wash-on. Baseline resting potential was measured from minutes 0-2. A 10 min CNO wash-on period occurred between minutes 2-12. A 20 min wash-off period occurred between minutes 12-32 (though no wash-off effect was seen). Error bars indicate mean ± SEM. Mixed-effects analysis. Fixed effect of treatment *F*_(1,21)_ = 9.76, *p* = 0.0051. Interaction of treatment × time *F*_(2,42)_ = 10.12, *p* = 0.003. *p < 0.01 Šídák multiple comparisons test. *n* = 6-17 cells. (***D***) Average number of aggressive interactions per treatment group across 8 d. *Mixed-effects analysis. Fixed effect of treatment *F*_(2,32)_ = 5.607, *p* = 0.0082. Main effect of time *F*_(3.622,115.4)_ = 5.147, *p* = 0.0011. Interaction of treatment and time *F*_(14,223)_ = 4.481, *p* < 0.001. *n* = 9-13 mice. (***E***) Average latency to attack per treatment group across 8 d. *Mixed-effects analysis. Interaction of treatment and time *F*_(14,217)_ = 1.959, *p* = 0.0221. *n* = 9-13 mice. (***F-I***) DeepLabCut-supported Random Forest predictions for listed behaviors. *Mixed-effects analysis. Interaction of treatment and time *F*_(2,14)_ = 3.842, *p* = 0.0467. Subset of animals used in panel ***C***, ***D***. *n* = 4-6 mice. Error bars indicate mean ± SEM.

We next sought to test whether ArcTRAP^+^ IGCs participate in intermale aggressive ramping. We hypothesized that inhibiting the ArcTRAP^+^ population may affect these behavioral outputs of chemosensory information processing. To test this hypothesis, we used inhibitory designer receptors exclusively activated by designer drugs (G_i_-DREADDs). A Cre-dependent AAV containing the G_i_-DREADD was injected into the AOB of male resident ArcTRAP mice. Control mice received a Cre-dependent AAV-mCherry. After 1 week of recovery from surgery, while also being solo-housed, the mice were TRAPed on day 0, activating CreERT2 and inducing expression of the G_i_-DREADD (or mCherry-only control). Following this initial resident–intruder interaction, residents were solo-housed for an additional week to allow the G_i_-DREADD receptor to be strongly expressed. Residents then underwent repeated resident–intruder interactions once per day for 7 d and received either a CNO or DMSO (vehicle) injection 20 min before behavior (Fig. 7*A*). Viral targeting resulted in high efficiency labeling of ArcTRAP^+^ cells (thresholded Manders coefficient for tdTomato pixels colocalized with mCherry pixels 0.853 ± 0.061, for mCherry pixels colocalized with tdTomato 0.8697 ± 0.059, *n* = 8 animals; Fig. 7*B*). To ensure that the G_i_-DREADD was having the anticipated hyperpolarizing effect on IGCs, we conducted a drug wash-on electrophysiology experiment in AOB slices (Fig. 7*C*). Resting membrane potential was recorded for a 32 min duration. Compared with IGCs expressing only mCherry, the G_i_-DREADD-expressing IGCs showed a significant hyperpolarization induced by the CNO wash-on, which persisted through the postdrug period (Fig. 7*C*), confirming that the G_i_-DREADDs were having the anticipated hyperpolarizing effect in IGCs.

After verifying that the G_i_-DREADD was actively inducing hyperpolarization in ArcTRAP^+^ IGCs during a CNO application, we assessed the effect of hyperpolarizing ArcTRAP^+^ IGCs on behavioral outcomes. We manually measured the number of resident-initiated aggressive interactions and latency to first attack, finding that mice receiving the AAV-mCherry and CNO injection displayed the same aggressive ramping behavior (Fig. 7*D*) as seen in unmanipulated residents (Fig. 6*B*). They also showed a similar decrease in latency to attack (Fig. 7*E*). Consistent with our hypothesis that ArcTRAP^+^ IGCs participate in ramping intermale aggression, we found that mice receiving the G_i_-DREADD and CNO injection lacked the ramping aggression seen in the mCherry control mice (Fig. 7*D*). They also displayed an increased latency to first attack (Fig. 7*E*). Mice receiving the G_i_-DREADD and DMSO control injection displayed moderately increased aggression, and increased latency to first attack; however, they were not at the same levels of the mCherry control (Fig. 7*D*,*E*). This may be explained through physiological changes induced solely by the expression of a G_i_-DREADD, in the absence of the activating drug, which has been shown in other studies (Saloman et al., 2016). Together, these data suggest that proper regulation of signaling in inhibitory IGCs is necessary for sex-typical intermale ramping aggression in the resident–intruder paradigm.

To determine whether these manipulations altered other aspects of social behavior, on a subset of experiments (for which we had top-down video recordings), we used the machine learning-based DeepLabCut software (Mathis et al., 2018) and random forest-based behavioral segmentation. This analysis showed that control mCherry/CNO mice had increased aggression on days 3 and 7 compared with DREADD/CNO mice (Fig. 7*F*). We did not see a difference in other relevant behaviors, such as exploration (time spent exploring the cage, sniffing bedding, etc.), approach (resident approaching intruder), or sniffing behavior (resident sniffing intruder) (Fig. 7*F–I*). We did observe a trend in the data suggesting that the DREADD/CNO group demonstrated higher nonaggressive sniffing behaviors than controls (Fig. 7*I*). Combined, these data show that, although expression and activation of G_i_-DREADDs strongly decrease the levels of aggression in resident mice, other nonaggressive behaviors are largely unchanged. This supports our hypothesis that ArcTRAP^+^ IGCs play a role in specifically regulating AOS-mediated aggressive behavior in the context of the resident–intruder paradigm.

## Discussion

Transgenic silencing and physical ablation of the vomeronasal organ established that the AOS is essential for several aspects of rodent social behavior (Stowers et al., 2002; Keller et al., 2006). The AOB itself has also been targeted for selective manipulation in the context of social behavior (Brennan et al., 1990; Otsuka et al., 2001; Kunkhyen et al., 2017). Despite the increased availability of cell type-specific transgenic tools for neuroscience, there are few tools that are selective enough to help delineate the functions of the several classes of neurons in the AOB (Cansler et al., 2017; Gao et al., 2017; Maksimova et al., 2019; Zhang and Meeks, 2020). The availability of transgenic tools for labeling and manipulating *Arc*-expressing neurons is especially helpful for studying AOB circuit function, as IGCs are the predominant cell type expressing this immediate-early gene in the AOB (Matsuoka et al., 2002; Cansler et al., 2017; Gao et al., 2017). Building on previous work that showed *Arc*-expressing IGCs demonstrate experience-dependent plasticity, we sought to understand whether this plasticity participated in longer-term AOS function, and whether the activity of these cells ultimately affects AOB signaling and the subsequent behavioral output.

ArcTRAP mice (Guenthner et al., 2013) provided an opportunity to investigate these questions. However, these and other tools that enable transient immediate-early gene-based labeling have limitations, requiring validation in a circuit and cell type-specific manner (Reijmers and Mayford, 2009; Guenthner et al., 2013; Allen et al., 2017). We investigated the utility of the ArcTRAP tool for studying AOB IGCs in the context of intermale territorial aggression, a context that has been shown previously to induce robust *Arc* expression in AOB IGCs (Cansler et al., 2017). This behavioral context is certainly not the only condition in which IGCs express *Arc* (Matsuoka et al., 2002; Gao et al., 2017), but it is a well-established AOS-mediated behavior, making it an attractive platform in which to perform initial testing and validation. TRAPing in this context was robust and cell type-specific (Fig. 1). Although TRAPing was strongest in social chemosensory conditions, we also observed increased tdTomato labeling in mice only receiving 4-OHT in their home cage (Fig. 1). There are several possible explanations for this observation. For example, in other brain regions, *Arc* expression is induced by environmental novelty (Cleland et al., 2017). Alternatively, it could be that sampling self chemosignals is sufficient to induce Arc-CreERT2 expression and recombination. It may also be that some AOB IGCs have spontaneous activity levels capable of driving sufficient *Arc* expression for ArcTRAPing. Regardless, these initial studies confirmed that ArcTRAP-based approaches selectively label AOB IGCs, and that the labeling reflects the chemosensory conditions present in the time window following 4-OHT injection as expected.

Two recent studies showed that *Arc*-expressing IGCs have increased excitability compared with other IGCs, and a major component of this effect was a change in the ability for *Arc*-expressing IGCs to maintain high spiking frequencies in the presence of strong depolarization (Cansler et al., 2017; Gao et al., 2017). We found that ArcTRAP^+^ IGCs maintain similar increases in excitability for multiple days following a single resident–intruder interaction (Fig. 2). As in previous studies, the increased intrinsic excitability of ArcTRAP^+^ IGCs was not accompanied by alterations in spontaneous EPSCs (Fig. 2*D*). The lack of obvious synaptic plasticity in these conditions does not preclude a subtle change in synaptic properties, but these experiments were not designed to tease apart such nuanced differences. Future studies would be needed to identify, for example, whether ArcTRAP^+^ IGCs are primed for future plasticity (“metaplasticity”), as has been seen in other *Arc*-expressing cells (Jakkamsetti et al., 2013). The observation that ArcTRAP^+^ IGCs maintain elevated excitability for several days after a social chemosensory encounter indicated that these cells are modified over time courses compatible with storing a chemosensory memory, a topic of much interest over the past several decades (for review, see Brennan, 2009). To determine whether *Arc*-expressing IGCs might play roles in chemosensory memory and/or social behavior plasticity, we proceeded to investigate *Arc* expression patterns after multiple social encounters.

In the hippocampus, well known for its role in spatial and episodic memory, RE to a familiar environment elicits *Arc* expression in a similar population of cells (Chawla et al., 2005). In contrast, exposure to a novel environment induces *Arc* expression in a mostly nonoverlapping neuronal population (Chawla et al., 2005). If *Arc*-expressing AOB IGC ensembles possess similar qualities in social chemosensory contexts, this could suggest the presence of an inhibitory “memory trace” at the first stage of sensory processing. Re-exposing a resident male to a familiar male intruder for 3 d after TRAPing induced highly overlapping *Arc* expression among ArcTRAP^+^ IGCs (Fig. 3). However, when a novel female mouse was introduced on the fourth day, *Arc* was expressed in a mostly nonoverlapping population of IGCs (Fig. 3). These data indicated that a similar ensemble of IGCs are reactivated, re-express *Arc* across multiple social interactions, and that the population of IGCs that express *Arc* reflect the social chemosensory environment. This also indicates that cells expressing *Arc* do not chronically express *Arc*, but that they transiently express *Arc* as a result of the specific intruder-induced AOB excitation.

It is not clear whether the re-expression of *Arc* across multiple days would sustain existing elevations of intrinsic excitability, or whether this would produce additional physiological effects. We therefore tested whether *Arc* re-expression produced new alterations to IGC physiology. If physiological changes in ArcTRAP^+^ IGCs scaled with each experience, we would expect to see an enhancement of physiological features that were affected after additional chemosensory encounters. We did find that increased IGC excitability persisted in ArcTRAP^+^ and ArcTRAP^–^ IGCs following multiple resident–intruder encounters. However, this increased excitability in RE experiments did not scale with repeated social encounters (Fig. 4). In both the SE and RE paradigms, we found that the excitability of ArcTRAP^+^ and ArcTRAP^–^ groups was statistically indistinguishable on day 7 (Fig. 5*F*). However, in the SE paradigm, ArcTRAP^+^ cells reduced their excitability to control levels by day 7 (Fig. 5*F*), suggesting that, in the absence of reactivation, the excitability effect waned. In contrast, in the RE paradigm, we found that the similarities between ArcTRAP^+^ and ArcTRAP^–^ maximum spiking frequency on day 7 were driven by an increase in excitability in the ArcTRAP^–^ group (Fig. 5*F*). We hypothesize that this may have been because of ArcTRAP^–^ IGCs (cells that were not TRAPed on day 0) being stimulated to express *Arc* between days 1 and 7. These cells would not express the tdTomato marker (because *Arc* expression occurred outside the 4-OHT time window), but would have expressed *Arc* in subsequent days, and therefore increased their excitability. This hypothesis is consistent with observations made during repeated exposure experiments involving ArcTRAP-d4eGFP mice (Fig. 3). In these experiments, we saw that a majority of ArcTRAP^+^ IGCs re-expressed *Arc*, but some ArcTRAP^+^ IGCs did not re-express *Arc*. Other IGCs expressed *Arc* on the last day, but not the first (Fig. 3). Future electrophysiology studies will be needed to determine whether more nuanced changes in IGC physiology, morphology, or gene expression occur as IGCs repeatedly express *Arc* across multiple social encounters.

IGCs are a heterogeneous population electrophysiologically, morphologically, and genetically (Larriva-Sahd, 2008; Cansler et al., 2017; Maksimova et al., 2019). This heterogeneity may have several sources, including cellular maturity; IGCs are continuously added to the AOB via adult neurogenesis in the subventricular zone and migration via the rostral migratory stream (Bonfanti et al., 1997). We explored the electrophysiological diversity of IGCs in the context of both SE and RE experiments to place ArcTRAP^+^ and ArcTRAP^–^ excitability changes in a broader context (Fig. 5). Clustering analysis identified high and low-excitability groups that were enriched in ArcTRAP^+^ and ArcTRAP^–^ IGCs, respectively (Fig. 5*B*). Using the high-excitability and low-excitability clusters as templates, we calculated a multidimensional vector that best separated these two populations. This allowed us to analyze the excitability of cells in our analysis in a way that incorporated all 23 physiological parameters, rather than just a single parameter (Fig. 5*C*,*D*). Despite their physiological heterogeneity, ArcTRAP^+^ IGCs project closer to the high-excitability phenotype that ArcTRAP^–^ IGCs in both SE and RE paradigms (Fig. 5*D*,*E*). The circuit functions of lower- and higher-excitability IGCs are not yet known, and future studies will be needed to determine how *Arc* expression impacts cellular maturity and longevity.

In observing the interactions between males during RE electrophysiology experiments, we noted that resident mice became more aggressive over the course of 7 d of RE (Fig. 6). Given that ArcTRAP^+^ IGCs have increased excitability across this same time course, we hypothesized that IGC activity may be playing a role in regulating these behavioral changes. When we inhibited the AOB ArcTRAP^+^ population with G_i_-DREADDs, we ablated the sex-typical aggressive ramping seen in repeated resident–intruder assays (Fig. 7). To our knowledge, this is the first report of an AOS-mediated behavioral effect driven by selective manipulation of an AOB interneuron subtype. The lack of ramping aggression in mice receiving G_i_-DREADDs and the DREADD agonist CNO could have been because of an off-target effect, for example, a reduction in main olfactory function, or other behavioral deficits. However, a DeepLabCut-based investigation of the microstructure of these encounters showed that other socially relevant behaviors (exploring, sniffing, approaching) were not affected by the manipulation of ArcTRAP^+^ IGCs (Fig. 7). In animals expressing the G_i_-DREADD but receiving vehicle injections, we observed an intermediate phenotype between controls and CNO-injected animals (Fig. 7). This may indicate that expression of G_i_-DREADDs causes disruption of these cells in the absence of the selective ligand. Agonist-independent effects of G_i_-DREADD expression on neurons have been reported elsewhere, and represent an important consideration for these tools (Saloman et al., 2016). Despite this possibility, the direction of the change is consistent with the overall conclusion of this experiment, which is that alteration of ArcTRAP^+^ IGC function reduces ramping aggression.

In these studies, we chose to selectively inhibit ArcTRAP^+^ IGCs, a manipulation likely to disinhibit AOB excitatory mitral cells. In female mice, AOB disinhibition causes failure of pregnancy maintenance, similar to the effects of a stranger male or its urine (Kaba and Keverne, 1988). If mitral cell hyperactivation increased the excitation by male chemosignals in the resident–intruder paradigm, one hypothesis would be that this would result in increased intermale aggressive behavior. However, the behavioral results indicate that this manipulation eliminated ramping aggression, an effect similar in nature to that of removing or silencing the vomeronasal organ itself (Wekesa and Lepri, 1994; Stowers et al., 2002). This suggests that *Arc*-expressing IGCs are necessary for the AOB to reliably transmit chemosensory information across repeated social encounters. Pharmacological blockade of GABA_A_ receptors in the AOB reduces discriminability of mitral cell responses to male and female urinary chemosignals (Hendrickson et al., 2008). It may be that hyperpolarizing ArcTRAP^+^ IGCs enhances responses to many MCs that would normally be minimally activated by nonmale cues, effectively “scrambling” the information produced by the AOB. Since hyperpolarizing ArcTRAP^+^ IGCs disrupted ramping aggression without entirely abolishing male–male aggression (Fig. 7*D*, day 0), it may also be the case that repeated resident–intruder interactions enhance the discriminability of male chemosignals compared with other conspecific cues, and that hyperpolarizing ArcTRAP^+^ cells circumvents this process. In future studies, it will be important to investigate these hypotheses, and to determine whether similar effects are seen with other AOS-mediated behaviors, such as mating and predator avoidance (Takahashi, 2014; Takahashi et al., 2018).

In conclusion, these studies demonstrate that a population of plastic interneurons in an early chemosensory circuit display physiological features consistent with simple memory formation. These neurons, when silenced, result in disruption of natural escalation of aggressive behavior between males, a well-known chemosensory-mediated behavior. Plasticity between excitatory and inhibitory interneurons is increasingly appreciated as critical for brain function, and these studies highlight that one of these roles is to support social behavior plasticity.
